# Identification of immunogenic cell death-related signature on prognosis and immunotherapy in kidney renal clear cell carcinoma

**DOI:** 10.3389/fimmu.2023.1207061

**Published:** 2023-08-18

**Authors:** Silin Jiang, Yuxiang Dong, Jun Wang, Xi Zhang, Wei Liu, Yong Wei, Hai Zhou, Luming Shen, Jian Yang, Qingyi Zhu

**Affiliations:** ^1^ Department of Urology, The Second Affiliated Hospital of Nanjing Medical University, Nanjing, China; ^2^ Department of Urology, Nanjing University of Chinese Medicine, Nanjing, China; ^3^ The State Key Lab of Reproductive; Department of Urology, The First Affiliated Hospital of Nanjing Medical University, Nanjing, China

**Keywords:** immunogenic cell death, kidney renal clear cell carcinoma, tumor immune microenvironment, prognostic signature, immunotherapy

## Abstract

**Background:**

Immunogenic cell death (ICD) is considered a particular cell death modality of regulated cell death (RCD) and plays a significant role in various cancers. The connection between kidney renal clear cell carcinoma (KIRC) and ICD remains to be thoroughly explored.

**Methods:**

We conducted a variety of bioinformatics analyses using R software, including cluster analysis, prognostic analysis, enrichment analysis and immune infiltration analysis. In addition, we performed Quantitative Real-time PCR to evaluate RNA levels of specific ICD genes. The proliferation was measured through Cell Counting Kit-8 (CCK-8) assay and colony-formation assay in RCC cell lines.

**Results:**

We determined two ICD subtypes through consensus clustering analysis. The two subtypes showed significantly different clinical outcomes, genomic alterations and tumor immune microenvironment. Moreover, we constructed the ICD prognostic signature based on TF, FOXP3, LY96, SLC7A11, HSP90AA1, UCN, IFNB1 and TLR3 and calculated the risk score for each patient. Kaplan-Meier survival analysis and ROC curve demonstrated that patients in the high-risk group had significantly poorer prognosis compared with the low-risk group. We then validated the signature through external cohort and further evaluated the relation between the signature and clinical features, tumor immune microenvironment and immunotherapy response. Given its critical role in ICD, we conducted further analysis on LY96. Our results indicated that downregulation of LY96 inhibited the proliferation ability of RCC cells.

**Conclusions:**

Our research revealed the underlying function of ICD in KIRC and screened out a potential biomarker, which provided a novel insight into individualized immunotherapy in KIRC.

## Introduction

Renal cancer is one of the most common malignant tumors around the world (1). Renal cell carcinoma (RCC) accounts for 90% of renal cancer and kidney renal clear cell carcinoma (KIRC) accounts for the majority of RCC (2). Although surgical operation brings a good prognosis to early-stage KIRC patients (3), advanced and metastatic KIRC still have poor clinical prognosis and outcome due to their insensitivity to radiotherapy or chemotherapy regimens (4). With the improved awareness of the role of immunological factor in tumor progression and prognosis, immunotherapy, especially checkpoint inhibitors, has become an important approach for unresectable KIRC (5, 6).

Immunogenic cell death (ICD) is a particular cell death modality of regulated cell death (RCD) (7, 8). Previous researches have indicated that ICD can induce adaptive immune response against the antigens of dead or dying tumor cells through damage-associated molecular patterns (DAMPs), which include ATP release, calreticulin exposure, and HMGB1 (high mobility group box 1) secretion (9, 10). The pivotal factor of cancer immunotherapy is how to avoid the immune escape of cancer (11). Specific immunogenic chemotherapy induces ICD to transform immune cold tumors into hot ones and increase the sensitivity of tumor cells to checkpoint inhibitors in several tumor cell lines (12). However, evidence of the effectiveness of this procedure is still lacking, which prompts us to explore the possibility of using ICD in clinical application.

In this study, we categorized patients on the premise of their expression of ICD genes and evaluated the difference in prognosis and immunotherapy response. We further identified several ICD biomarkers and constructed a scoring signature in which risk score was prominently associated with clinical features and tumor progression. Eventually, we predicted several drugs with high sensitivity to high-risk patients. We furthermore speculated that LY96 may serve as a potential novel therapeutic target and we verified the findings by experiments. Our results provided new clues for the development of tumor immunotherapy for KIRC.

## Materials and methods

### Retrieval of ICD genes

We obtained 1,736 ICD-related genes using the keyword “immunogenic cell death” in the GeneCards database (https://www.genecards.org/). At the same time, we summarized 171 ICD-related genes from relevant literature (13, 14). Then, the intersection of two gene sets yielded 73 genes that were considered as ICD key genes and included in our research.

### Acquisition and preprocessing of data

The TPM transcriptome data that involved 541 tumor samples and 72 normal samples and matched clinical data of KIRC were obtained from the TCGA database (https://portal.gdc.cancer.gov/). The E-MTAB-1980 dataset (https://www.ebi.ac.uk/arrayexpress/experiments/E-MTAB-1980/) was selected as external validation cohort, which comprised RNA sequencing data and clinical information of 101 KIRC samples. Samples without survival data were removed from the cohort.

### Differentially expressed ICD genes and protein–protein interaction network

Differentially expressed ICD genes (DEIGs) were identified by the “limma” R package (15). The protein–protein interactions (PPIs) among DEIGs were constructed using the Search Tool for the Retrieval of Interacting Genes (STRING) database (https://string-db.org/). Cytoscape v3.9.1 was used to draw the network (16). MCODE was a plugin of Cytoscape, which we conducted to identify highly interconnected functional cluster.

### Construction of ICD-related subtypes and functional enrichment analyses

The R package “ConsensusClusterPlus” was performed to identify ICD molecular subtypes. The maximum subtypes were set at nine and the maximum number of iterations was set to 1,000 to guarantee the reliability of statistical analysis. Samples were clustered into two subtypes according to the result. Differentially expressed genes (DEGs) between two ICD subtypes were identified with cutoffs of |log2 fold change (FC)| > 1 and false discovery rate (FDR)< 0.05 for functional enrichment analyses. Gene Ontology (GO) and Kyoto Encyclopedia of Genes and Genomes (KEGG) analyses were implemented to predict proper biological functions and pathways of DEGs between ICD subtypes through the “ClusterProfile” package. Gene set enrichment analysis (GSEA) was also performed to investigate proper mechanism of actions of DEGs *via* GSEA version 4.1.0 (http://software.broadinstitute.org/gsea/). KEGG, Hallmark, and Reactome gene sets were downloaded from the Molecular Signature Database (MSigDB, https://www.gsea-msigdb.org/gsea/downloads.jsp). The minimum gene set was set as 5 and the maximum gene was set as 5,000 based on the gene expression profile and phenotypic grouping. Each gene set was repeatedly permutated 1,000 times for each analysis. *p*-value< 0.05 was considered to be statistically significant.

### Comparison of genomic alterations of different ICD subtypes

Somatic mutation data of KIRC patients were downloaded from the TCGA database in “maf” format. Waterfall plots were plotted by the “Maftools” R package to visualize and summarize gene mutation. We further downloaded the segmented copy number variation (SCNV) data of KIRC from the GDC portal using the “TCGAbiolinks” R package for somatic copy number analysis according to a previous study (17). The alteration of gene copy number and GISTIC score for each sample were analyzed through GISTIC 2.0 software (https://cloud.genepattern.org/). We also calculated the burden of copy number loss or gain on the basis of total number of genes with copy number changes at focal and arm levels for further comparison between two ICD subtypes (18).

### Tumor immune microenvironment of ICD subtypes

The ESTIMATE algorithm was conducted to evaluate the tumor immune microenvironment (TME) of KIRC patients (19). The ESTIMATE algorithm calculated the stromal and immune score to predict the infiltration of matrix and immune cells. The CIBERSORT algorithm was applied to convert the gene expression data into expression of 22 immune cell types (20). The immune cell type with low expression was removed. By analyzing the correlation and difference of immune cell types between two subtypes, we mapped the correlation heatmap and multiple-group barplot to visualize the results. Furthermore, we analyzed the difference of HLA and checkpoint genes expression between the two subtypes. The HLA and checkpoint genes were acquired from a previous study (21).

### Construction and validation of ICD prognostic signature

Univariate Cox regression was performed to screen out prognosis-related ICD genes with the criteria *p*< 0.05 of training set. Dimension reduction was carried out through the supervised regression random forest algorithm of the “randomForestSRC” package (ntree = 1,000) (22). The top 10 significant genes were selected for further multivariate Cox regression. ICD risk score was calculated by the following formula:


$$\text{Risk score}=\sum_{i=1}^{N}\alpha_{i}x_{i}$$

*N*, *α*, and *x* represent the number of selected genes, coefficient, and expression value. Patients in the training and validation set were divided into two groups according to ICD risk score. Kaplan–Meier (KM) survival curve and ROC curve were used on both the training set and validation set to assess the reliability of the ICD Prognostic Signature. Area under the curve (AUC) was used to quantify the ROC curve. We then visualized the clinical features of two risk groups by a heatmap. Variation analyses of clinical factors between different risk groups and correlation analyses focused on ICD risk score and clinical factors were also conducted. Univariate and multivariate Cox regression analyses were used to figure out independent prognostic factors. A nomogram was plotted based on the R package “NomogramEX” (23) and proportional hazards assumption was examined. Calibration curves of 1, 3, and 5 years were plotted to assess the nomogram.

### Immunotherapy response prediction

TIDE (Tumor Immune Dysfunction and Exclusion) was an algorithm that integrated the characteristics of T-cell dysfunction and T-cell exclusion to predict immunotherapy response in tumor patients. The TIDE webserver (http://tide.dfci.harvard.edu/) was used to analyze the normalized expression data, and assigned a TIDE score to each patient where >0 was determined as no responder and<0 was determined as responder. The Subclass Mapping (SubMap) method was also put into use to predict the response of different groups to anti-PD-1 and anti-CTLA4 immunotherapy. In this analysis, we compared the expression profile of the two ICD risk groups we defined with another published dataset containing 47 patients with melanoma that responded to immunotherapies (24).

### Connectivity map analysis

The Cmap website (https://clue.io/) provides a connectivity map analysis to predict potential useful small molecular drugs using the 150 most significant up- and downregulated DEGs between two risk groups. All 300 DEGs included in our analysis were identified using the “limma” R package and showed a significant difference with the criterion of *p*< 0.05. The inclusion criterion for determining potential useful small molecular drugs was the absolute value of Cmap score greater than 90.

### Cell culture and quantitative real-time PCR

Human RCC cell lines, including 786-O and 769-P, and the human renal tubular epithelial immortalized cell line HK-2 were obtained from the Cell Bank of the Chinese Academy of Sciences (Shanghai, China). 786-O and 769-P cells were cultured in Roswell Park Memorial Institute medium (RPMI-1640; Gibco) and HK-2 was cultured in DMEM/F-12 (Gibco). All these cells were maintained in medium supplemented with 10% fetal bovine serum (Gibco) and 1% penicillin/streptomycin (Thermo Fisher) at 37°C in a 5% humidified CO_2_ atmosphere.

A total of nine paired fresh-frozen KIRC tissues and normal tissues were obtained from patients diagnosed with KIRC at The Second Affiliated Hospital of Nanjing Medical University.

The total RNAs were isolated from tissues or cells using Trizol reagent (Invitrogen Life Technologies) according to the manufacturer’s instructions. The quantity and quality of the extracted total RNA were assessed by using a NanoDrop 2000c spectrophotometer (Thermo Scientific). The total RNA was reverse-transcribed using HiScript III All-in-one RT SuperMix Perfect for qPCR (Vazyme; R333). Quantitative real-time PCR (qRT-PCR) was performed with Taq Pro Universal SYBR qPCR Master Mix (Vazyme; Q712-02) using a CFX96 Touch Real-Time PCR Detection System (Bio-Rad). Beta-actin was used as an internal control, and the relative expression level for genes was calculated by the 2^−ΔΔCt^ method. The primers used for qRT-PCR are listed in **Table S3**.

### Cell transfection

For transfection, cells were seeded in six-well plates and grown to 40%–60% confluence by the time of transfection. Small interfering RNA (siRNA) and its negative control reagents were purchased from GenePharma Company. siRNAs were transfected with Lipofectamine™ 3000 reagent (Invitrogen, USA) according to the manufacturer’s instructions. Target sequences of the siRNAs are shown in **Table S4**.

### Cell Counting Kit-8 assay

Cell proliferation was measured by using the Cell Counting Kit-8 (CCK-8) (Vazyme; A311-01) according to the manufacturer’s instructions. Briefly, cells were seeded onto plastic 96-well plates at an initial density of 2 × 10^3^ cells/well. Then, CCK8 solution was added to each well at the indicated times and incubated for an additional 2 h at 37°C. Thereafter, OD_450_ values were measured.

### Colony formation assay

The clonogenic potential of transfected or infected cells was evaluated by plate colony formation assay. Cells were seeded onto plastic six-well plates at an initial density of 1 × 10^3^ cells/well in appropriate growth media and incubated for 2 weeks. The cells were fixed with 4% paraformaldehyde, and stained with Crystal Violet Staining solution (Beyotime; C0121). The stained cell colonies were counted and analyzed.

### Statistical analysis

Statistical analysis and figures were performed using R software v4.1.0 and GraphPad Prism 8 (San Diego, USA). Spearman analysis was performed to calculate correlation coefficients. Chi-square test was used for categorical data. The association between clinicopathologic data and expression profile was estimated by the Wilcoxon rank test and logistic regression. All results with *p*-value< 0.05 were considered statistically significant. The pheatmap and ggplot2 R packages were engaged for the mapping. KM survival and ROC curve based on survival and timeROC packages were performed to assess survival outcomes. Sangerbox (www.sangerbox.com) was used to improve the quality of figure. *, **, ***, and **** represent *p*< 0.05, *p*< 0.01, *p*< 0.001, and *p*< 0.0001, respectively.

## Results

### Identification of differentially expressed ICD genes and the protein–protein interaction network

**Figure 1:**
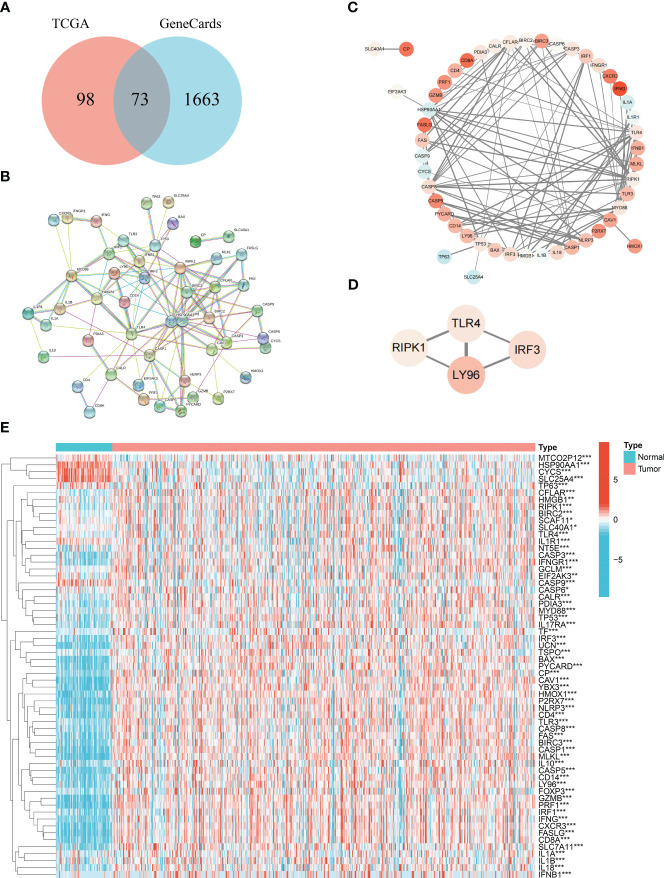
Acquisition of common ICD genes. **(A)** Venn diagram of the 73 common ICD genes. **(B)** Protein–protein interactions among the 73 common ICD genes. **(C)** Visualization of the PPI network conducted on Cytoscape. **(D)** Visualization of the functional subnet module. **(E)** Heatmap of differentially expressed ICD genes between normal and tumor samples in KIRC. * represents p< 0.05, ** represents p< 0.01, *** represents p< 0.001.

From previous literatures and GeneCards database (25), 73 common genes were considered as ICD core gene (**Table S1**). Subsequently, the R package “limma” was applied to identify DEIGs (**Figure 1A**). A total of 61 DEIGs, namely, 52 upregulated and 9 downregulated genes, were screened out. A heatmap was used for visualization of the expression (**Figure 1E**). The PPI network of DEIGs was retrieved using the STRING database (**Figure 1B**) and visualized by the Cytoscape software (**Figure 1C**). Functional key subnetwork analysis was performed through the MCODE algorithm, consisting of the following modules: LY96, TLR4, IRF3, and RIPK1, which was considered as a significant module with a high MCODE score (**Figure 1D**).

### Generation of two ICD subtypes through consensus clustering

**Figure 2:**
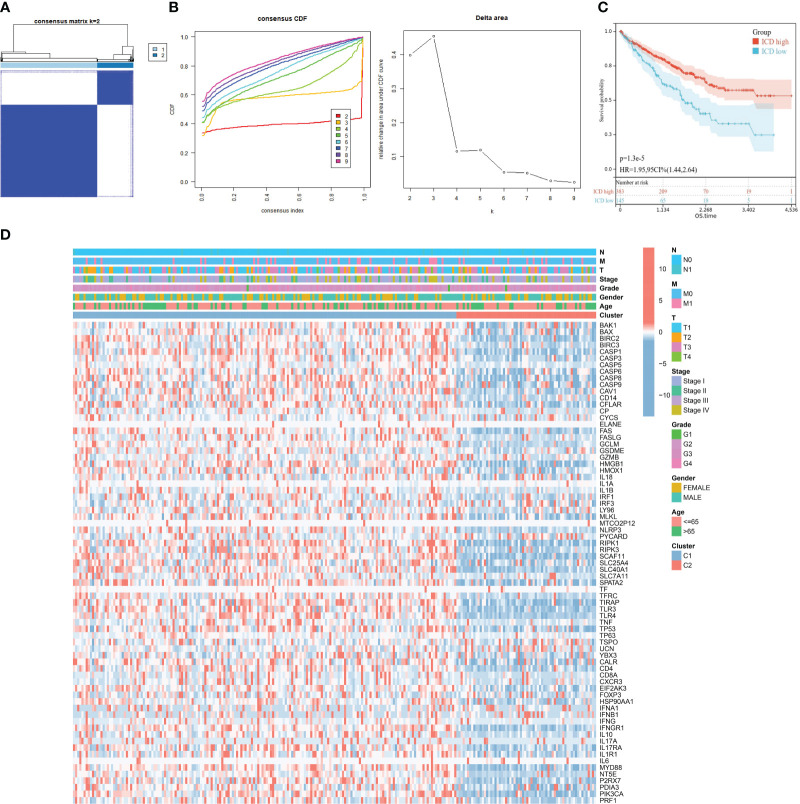
Construction of two ICD subtypes through consensus clustering. **(A)** Heatmap exhibits consensus clustering result for *k* = 2. **(B)** Consensus clustering cumulative distribution function (CDF) and delta area under the CDF curve for *k* = 2 to *k* = 9. **(C)** Kaplan–Meier curves of OS in ICD-high and ICD-low subtypes. **(D)** Heatmap of 73 ICD gene expression and clinical factors in different subtypes. Corresponding feature names are shown at the right of the heatmap.

To further reveal the relationship between expression of DEIGs and KIRC, we utilized the “ConsensusClusterPlus” R package to classify molecular subtype with KIRC patients according to the expression levels of DEIGs. Samples were clustered into two clusters after K-means clustering (**Figures 2A, B**). Then, KM survival analysis indicated that patients in the ICD-low subtype showed dismal prognosis compared with patients in the ICD-high subtype (**Figure 2C**). Furthermore, as displayed in **Figure 2D**, the genomic expression of ICD genes was compared in two clusters. Cluster C1 (n=383) was considered as ICD-high subtype for exhibiting a higher expression of ICD genes while cluster C2 (*n* = 145) was considered as ICD-low subtype. Differences of clinical features between the two distinct subtypes were also plotted for visualization in **Figure 2D**.

### Functional enrichment analyses

**Figure 3:**
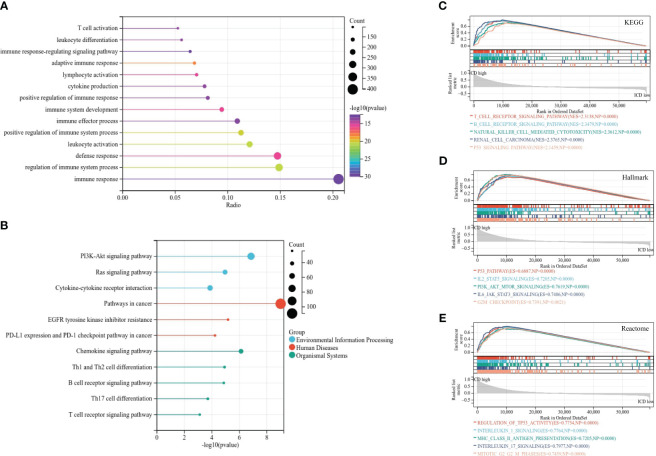
Functional enrichment analysis of differentially expressed genes in different subtypes. **(A, B)** Lollipop plot of GO **(A)** and KEGG **(B)** signaling pathway enrichment analysis. **(C–E)** GSEA analyses based on KEGG **(C)**, Hallmark **(D)**, and Reactome **(E)** gene sets.

In order to investigate the potential molecular mechanism and biological activity of ICD subtypes, subtype-related DEGs were figured out for functional enrichment analysis for GO and KEGG analysis. GO analysis demonstrated that DEGs were mainly involved in immune response, regulation of immune system process, defense response, and leukocyte activation (**Figure 3A**). KEGG analysis revealed that DEGs were mainly enriched in cancer-associated pathways, including the PI3K-Akt signaling pathway, EGFR tyrosine kinase inhibitor resistance, PD-L1 expression and the PD-1 checkpoint pathway in cancer, and the chemokine signaling pathway (**Figure 3B**), implying that immunogenic cell death acts as a crucial factor in the progression of RCC. Moreover, GSEA based on KEGG, Hallmark, and Reactome gene sets was used for further exploration. The results suggested that immunity and cancer-related pathways were highly concentrated in the ICD-high subtype, including the T- and B-cell receptor signaling pathway, the p53 signaling pathway, IL2-STAT5 signaling, and interleukin 1 and 17 signaling (**Figures 3C–E**).

### Genomic alterations of different ICD subtypes

**Figure 4:**
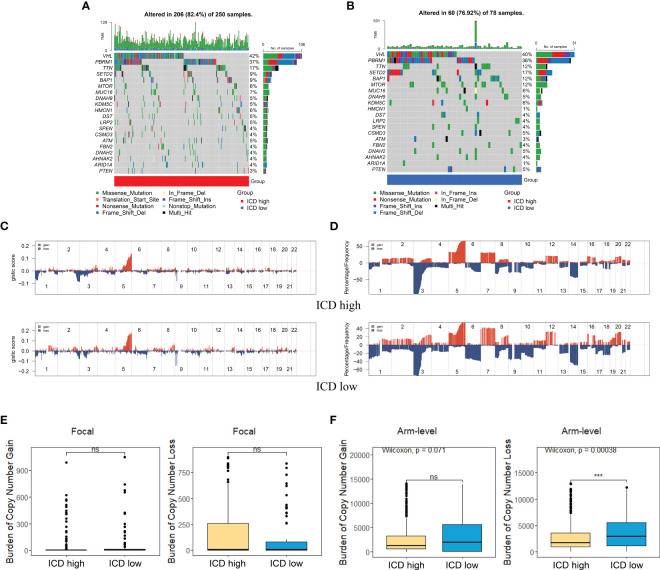
Comparison of genomic alternations between different subtypes. **(A, B)** Oncoprint display of the 10 most frequently mutated genes in the ICD-high subtype **(A)** and ICD-low subtype **(B)**. **(C, D)** Comparison of GISTIC score **(C)** and gain/loss percentage **(D)** of copy number profiles between different subtypes. **(E)** Focal level of CNV burden between two subtypes. **(F)** ICD-low subtype showed a higher arm level of CNV burden. *** represents p< 0.001.

The somatic mutation landscape was also analyzed in two subtypes (**Figures 4A, B**). Although VHL, PBRM1, TTN, and SETD2 were the most frequent mutations, the relative frequency varied among different subtypes. We then analyzed the GISTIC scores and copy number gain/loss percentage in the ICD-high and -low group. The result revealed that the ICD-low subtype was more likely to have a higher GISTIC score (**Figure 4C**) and copy number gain/loss percentage (**Figure 4D**). The burden of copy number gain and loss in the ICD-high group was decreased compared with the ICD-low group at arm level while there was no remarkable difference at focal level (**Figures 4E, F**). It appeared that arm level copy number alterations mainly contributed to the difference in ICD expression level.

### Assessment of tumor immune microenvironment and checkpoints in distinct subtypes

**Figure 5:**
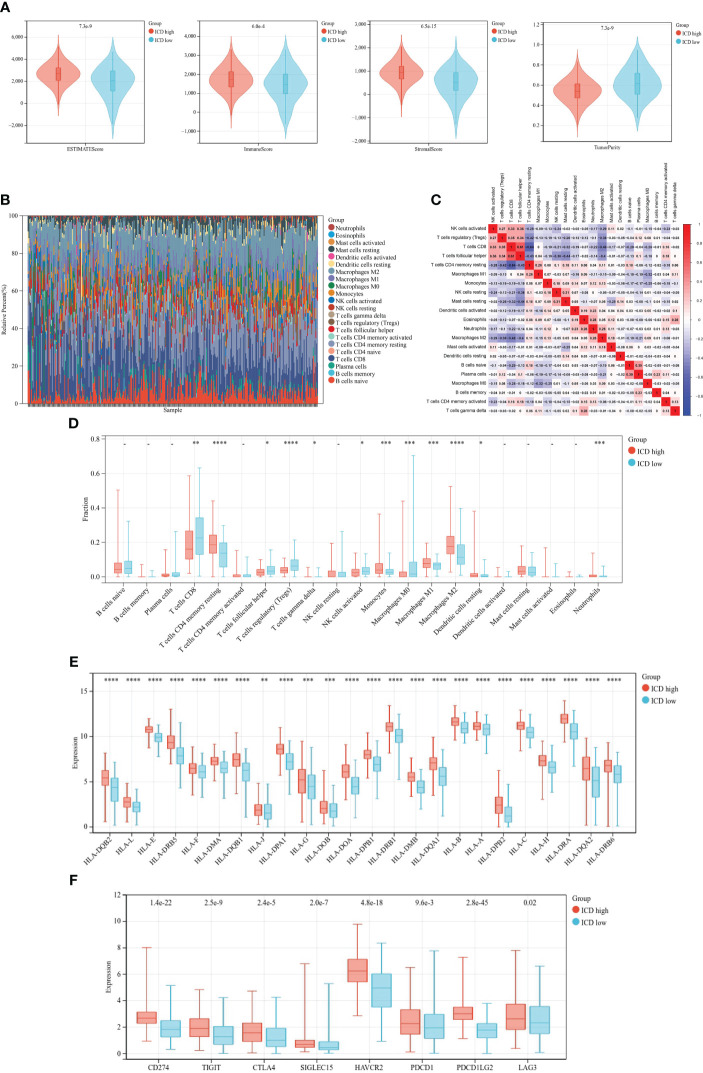
Immune landscape of different ICD subtypes. **(A)** Violin plots of ESTIMATE, immune, stromal scores, and tumor purity of ICD-high and -low subtypes. **(B)** Relative proportion of immune infiltration. **(C)** Correlation heatmap of 21 immune cells. **(D–F)** Box plots of differential expression of 21 immune cells **(D)**, HLA genes **(E)**, and immune checkpoints **(F)** between ICD-high and -low subtypes. *, **, ***, and **** represent *p*< 0.05, *p*< 0.01, *p*< 0.001, and *p*< 0.0001, respectively.

Accumulating evidence revealed that ICD had significant correlation with antitumor immunity. In our research, we analyzed the tumor immune microenvironment of two subtypes and discriminated immune-related characteristics between two subtypes. We first calculated the TME status using the ESTIMATE algorithm. As depicted in **Figure 5A**, the stromal score, immune score, and ESTIMATE score (*p*< 0.05) were significantly higher in the ICD-high subtype than those in the ICD-low subtype while tumor purity was the opposite.

Then, we calculated the fraction of 22 kinds of tumor-infiltrating immune cells (TIICs) through the CIBERSORT algorithm and removed the low-expression cell line. Grouping histogram showed the distribution of TIICs (**Figure 5B**). Macrophages and T cells accounted the most for the total. Pearson’s correlation was performed to analyze TIIC correlation (**Figure 5C**). We next examined immune cell infiltration to assess differences in the immune context of the tumor immune microenvironment between two subtypes. The ICD-high subtype showed high infiltration of CD8 T cells, activated CD4 memory T cells, follicular helper T cells, regulatory T cells (Tregs), and M0 macrophages, while the ICD-high subtype was characterized by high infiltration of resting CD memory T cells, monocytes, M1 and M2 macrophages, and resting dendritic cells (**Figure 5D**). Meanwhile, the expressions of HLA genes and immune checkpoint genes were different among the distinct subtypes. The result suggested that HLA genes (**Figure 5E**) and checkpoint genes (**Figure 5F**) were markedly higher in the ICD-high subtype.

### Construction and validation of the ICD prognostic signature

**Figure 6:**
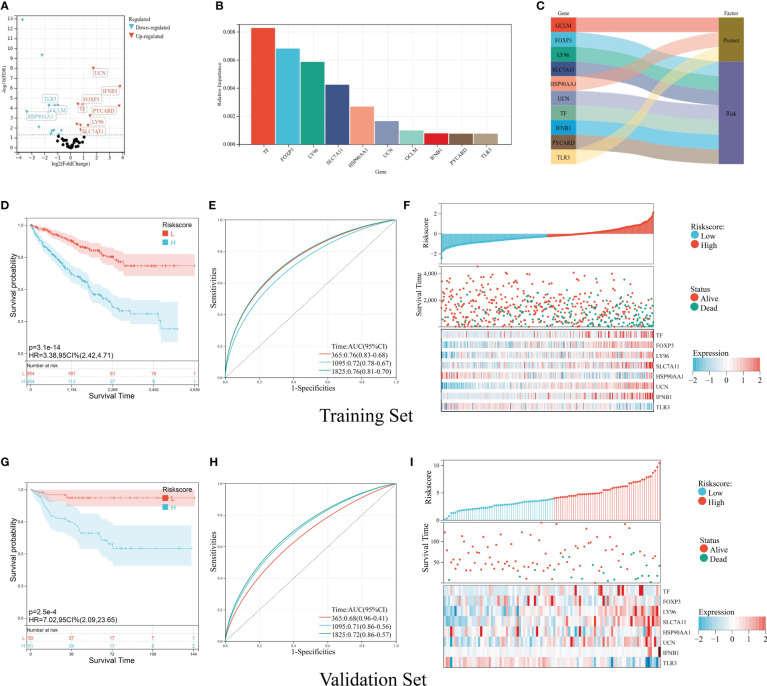
Construction and validation of the ICD prognostic signature. **(A)** Volcano plot of prognosis-related ICD genes preliminarily identified by univariate Cox analysis with the screening criteria *p*< 0.05. The red icons represent risk factors (HR > 1), and the blue icons represent protective factors (HR< 1). **(B)** The top 10 important ICD genes based on the relative importance calculated by random forest algorithm. **(C)** Sankey diagram demonstrated the prognosis effect of top 10 important ICD genes. **(D-F)** Kaplan–Meier curve of OS prognosis **(D)**, timeROC plot **(E)**, and risk plot including risk score distribution, survival status, and heatmap of eight signature genes **(F)** in the training set. **(G, I)** Kaplan–Meier curve of OS prognosis **(G)**, timeROC plot **(H)**, and risk plot including risk score distribution, survival status, and heatmap of eight signature genes **(I)** in the validation set.

For the purpose of predicting the prognosis accurately and credibly, we constructed an ICD prognostic signature based on supervised regression random forest algorithm. The top 10 significant genes—7 risk genes and 3 protect genes—were screened out (**Figures 6A-C**). KM analysis were carried out on the 1,023 combinations of the top 10 genes (**Table S2**). We selected the combination with the lowest *p*-value of KM analysis as ICD prognostic signature containing TF, FOXP3, LY96, SLC7A11, HSP90AA1, UCN, IFNB1, and TLR3. The ICD risk score was calculated as follows: ICD score = (0.10917254 * TF) + (0.16458303 * FOXP3) + (0.90393805 * LY96) + (0.50920311 * SLC7A11) + (−0.88020896 * HSP90AA1) + (0.99872821 * UCN) + (1.28833498 * IFNB1) + (−0.78540411 * TLR3). We allocated patients into high-risk and low-risk group according to their ICD risk score. KM survival analysis was performed to determine the overall survival (OS) time between different risk groups and ROC curve quantifying by AUC was utilized to examine prognosis on the training set (TCGA cohort) and validation set (E-MTAB-1980 cohort). According to our results, patients with low ICD risk score demonstrated a prominent survival benefit in both training set and validation set (**Figures 6D, E**). The AUC curves showed that ICD risk score had an acceptable prognostic value for KIRC patients. The AUC values for predicting 1-, 3- and 5-year OS in the training set were 0.76, 0.72, and 0.76, respectively, and those in the validation set were 0.68, 0.71, and 0.72 (**Figures 6G, H**). Additionally, expressions of survival status and heatmap of each set were also presented (**Figures 6F, I**).

### Clinical features of the prognostic ICD risk signature

**Figure 7:**
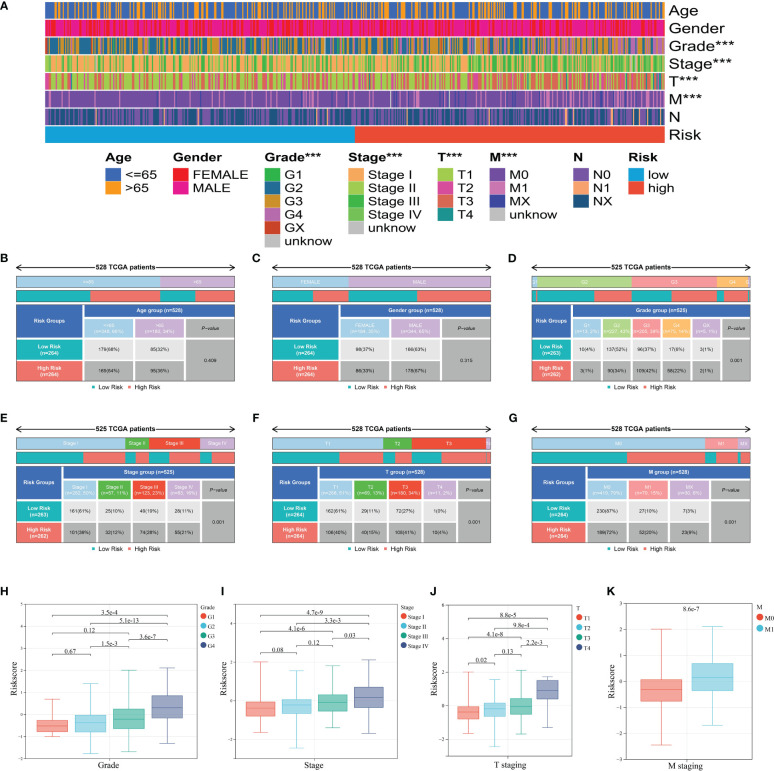
Clinical relevance of the ICD prognostic signature. **(A)** Heatmap of clinical factors in different risk groups. **(B-G)** Clinical differences between high and low risk groups including age **(B)**, gender **(C)**, grade **(D)**, stage **(E)**, T staging **(F)**, and M staging **(G)**. **(H-K)** ICD score differences between groups of grade **(H)**, stage **(I)**, T staging **(J)**, and M staging **(K)**. *** represents p< 0.001.

After clinical information analysis, we first drew a heatmap to illustrate the difference between two risk groups (**Figure 7A**). Then, Chi-square test was performed to evaluate the clinical difference between two risk groups. Grade, stage, T staging, and M staging were considered to have a significant difference between the high- and low-risk group whereas age and gender had no difference (**Figures 7B-G**). Meanwhile, we further analyzed the correlation of ICD risk score and four diverse clinical parameters. The boxplots showed the substantially elevated ICD risk score in the higher grade, stage, T staging, and M staging according to the *p*-value of difference analysis between the groups (**Figures 7H-K**). Thus, it was surprising that the value of ICD risk score had the capability to assess tumor progression.

### Establishment of nomogram to predict patient prognosis

**Figure 8:**
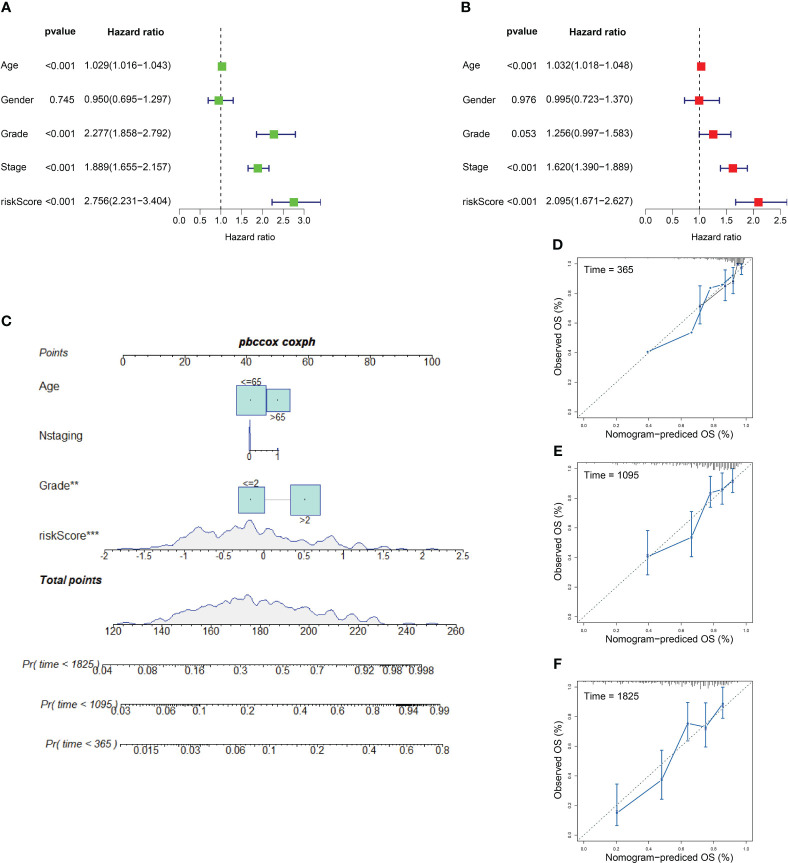
Independent prognostic factors and nomogram model. **(A, B)** Outcomes of univariate prognostic analysis **(A)** and multivariate prognostic analysis **(B)**. **(C)** Nomogram for evaluating the possibility of KIRC patients mortality at 1, 3, and 5 years. **(D–F)** Calibration for assessing the conformity between nomogram OS and observed OS at 1 year **(D)**, 3 years **(E)**, and 5 years **(F)**. ** represents p< 0.01, *** represents p< 0.001.

We applied univariate and multivariate Cox regression analyses to explore independent prognostic factors. Clinicopathologic features including age, gender, grade, and stage with ICD risk score were displayed in the training set, which confirmed that ICD risk score was an independent prognostic factor of KIRC (univariate Cox: HR: 2.758, 95% CI: 2.231–3.404, *p*-value< 0.001; multivariate Cox: HR: 2.095, 95% CI: 1.671–2.827, *p*-value< 0.001, respectively) (**Figures 8A, B**). Owing to the high correlation between ICD risk score and prognosis, clinical parameters including age, N staging, and grade together with ICD risk score were incorporated to construct a nomogram. All features in the nomogram met the standard of *p*-value of proportional hazards assumption greater than 0.05. The nomogram was utilized to estimate 1-, 3-, and 5-year OS for KIRC patients (**Figure 8C**). As shown in **Figures 8D–F**, calibration curves of 1, 3, and 5 years were established to evaluate the performance of nomogram and presented great accuracy between actual observations and predicted values.

### Relation between ICD signature and tumor immune microenvironment

**Figure 9:**
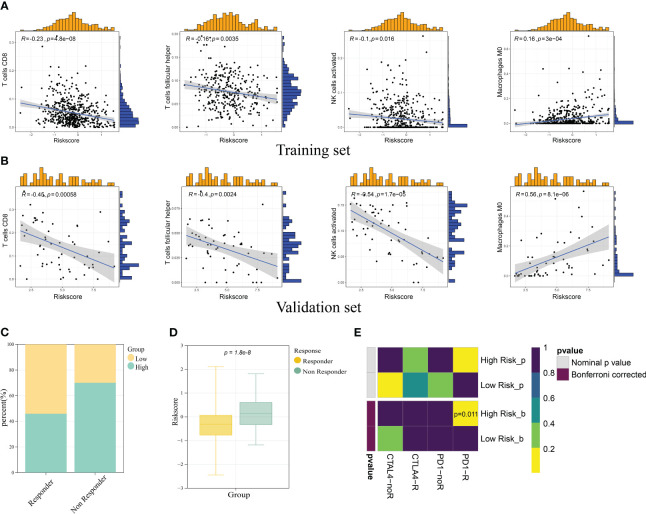
Correlation of ICD prognostic signature with immune cells and immunotherapy responses. Scatter plots revealed the correlation between risk score and infiltration of CD8 T cells, follicular helper T cells, activated NK cells, and M0 macrophages in the training set **(A)** and validation set **(B)**. **(C)** The immunotherapy responders had a higher percentage in the low risk group. **(D)** The immunotherapy responders had a lower risk score. **(E)** Submap analysis manifested the sensitivity of patients in different risk groups to PD1 and CTLA4 therapy.

Based on the findings above, we had confirmed the potential role of ICD in antitumor immune response. The relation between ICD risk score and TIICs was scrutinized. The results demonstrated that patients with elevated ICD risk score exhibited a negative correlation with CD8 T cells, follicular helper T cells, activated NK cells, and a positive correlation with M0 macrophages (**Figure 9A**). The validation cohort showed the same tendency (**Figure 9B**).

To investigate the role of ICD risk score on response to immunotherapy, we used TIDE (http://tide.dfci.harvard.edu) analysis to quantify the rate of response to TIDE score for each patient. The results showed that the high-risk group had a higher percent of non-responder patients (**Figure 9C**). Notably, immunotherapy responder patients showed a lower ICD score compared with non-responder patients (*p*-value< 0.05) (**Figure 9D**). In addition to TIDE prediction, we also compared the expression profile of two risk groups with a published dataset containing 47 patients with melanoma that responded to immunotherapies. As for our result, the high-risk group was more conceivable to respond to anti-PD-1 therapy with the Bonferroni-corrected *p*-value of 0.011 (**Figure 9E**).

### Prediction of small molecular drug

We employed the Connectivity Map (CMap) tool, which was widely used to discover potential small molecular drugs, with 150 up- and downregulated DEGs between two risk groups. We finally identified 12 candidate small molecular drugs with absolute CMap score > 90, namely, fostamatinib, YC-1, NM-PP1, torin-2, tipifarnib-P2, apigenin, SB-431542, cycloheximide, amonafide, linifanib, piperacillin, and ochratoxin-a (**Table 1**).

**Table 1:** Candidate small molecular drugs analyzed by CMap tools.

Name	Score	MOA	Target
Fostamatinib	97.92	SYK inhibitor	SYK, FLT3, RET
YC-1	96.26	Guanylyl cyclase activator	HIF1A, GUCY1A2, GUCY1A3, GUCY1B3
NM-PP1	94.11	Mutant kinase inhibitor	CAMK2A, LCK, MAPK8, PRKACA, RIPK2, SRC
Torin-2	93.59	MTOR inhibitor	MTOR
Tipifarnib-P2	93.37	Farnesyltransferase inhibitor	FNTA, FNTB
Apigenin	90.81	Casein kinase inhibitor, cell proliferation inhibitor, cytochrome P450 inhibitor	AKR1B1, AR, CDK6, CFTR, CYP19A1, CYP1A2, CYP1B1, HSD17B1, MAOA, ODC1, XDH
SB-431542	90.08	TGF beta receptor inhibitor	TGFBR1, ACVR1C, ACVR1B
Cycloheximide	−93.2	Protein synthesis inhibitor	GSK3B, RPL3
Amonafide	−95.98	Topoisomerase inhibitor	TOP2A, TOP2B
Linifanib	−96.26	PDGFR receptor inhibitor, VEGFR inhibitor	CSF1R, KDR, PDGFRB, FLT1, FLT3, FLT4, CSF1, KIT, PDGFRA, RET, TEK
Piperacillin	−97.5	Bacterial cell wall synthesis inhibitor	none
Ochratoxin-a	−97.88	Phenylalanyl tRNA synthetase inhibitor	SLC22A6

### LY96 promotes the proliferation of KIRC *in vitro*

**Figure 10:**
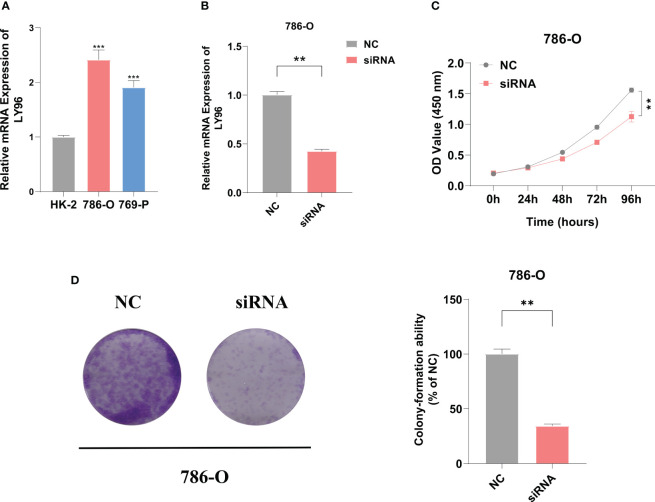
LY96 promotes the proliferation of ccRCC *in vitro*. **(A)** qRT-PCR verified the expression level of LY96 in RCC cell lines. **(B)** qRT-PCR analysis of LY96 mRNA in 786-O cells treated with negative control (NC) or LY96 siRNA. **(C)** CCK-8 was performed to determine the proliferation abilities of 786-O cells treated with negative control (NC) or LY96 siRNAs. **(D)** Colony formation was performed to determine the proliferation abilities of 786-O cells treated with negative control (NC) or LY96 siRNAs. ** represents p< 0.01, *** represents p< 0.001.

The eight ICD signature genes’ expression was analyzed by qRT-PCR in nine pairs of KIRC and adjacent tissues (**Figure S1**). We measured the mRNA expression of LY96 in human renal cortex proximal convoluted tubular epithelial cell (HK-2) and two human KIRC cell lines (786-0 and 769-P), and the highest expression was found in 786-O (**Figure 10A**). To evaluate the biological roles of LY96 in KIRC, small interfering RNA (siRNA) that specifically target LY96 was designed. According to the expression of LY96 in different cell lines, siRNA-LY96 was transfected into 786-O. The knockdown efficiency was confirmed by qRT-PCR analyses, which showed that more than 50% LY96 was knockdown. As shown in **Figure 10B**, the expression levels of LY96 were significantly decreased in siRNA-infected 786-O cells compared to negative control (NC) cells. CCK-8 and colony formation experiments demonstrated that downregulation of LY96 inhibited the proliferation ability of 786-O cells (**Figures 10C, D**).

## Discussion

Cancer immunotherapy has made a revolution in cancer treatment through establishing a connection between the human immune system and cancer (26). Various types of immunotherapies, including cellular or antibody therapy (27), immune checkpoint therapy (28), CAR T-cell therapy (29), and cancer vaccination (30), have been applied to KIRC patients (31). ICD is a kind of RCD and considered sufficient to activate an adaptive immune response (32, 33). The mechanism of action encompasses the release of DAMPs, which can be recognized by innate pattern recognition receptors (PRRs) from dying tumor cells, which results in tumor-specific immune response (34). In addition, numerous drugs in other kinds of radiation therapy, chemotherapy, or immunotherapy have the potential to augment ICD (35). Overall, we believed that ICD therapy together with other therapies will be greatly beneficial for cancer treatment.

Our research identified 73 core ICD genes through searching previous studies and public databases. Consensus clustering analysis was applied to split patients into two subtypes based on ICD gene expression. Our research revealed that the ICD-low subtype tended to have a favorable clinical outcome. We then screened the DEGs between high and low subtypes of ICD and used them in biological function and pathway enrichment analyses. Based on the results of enrichment analysis, DEGs were mainly enriched in biological functions such as immune response, regulation of immune system process, defense response and leukocyte activation, and pathways associated with immunity and cancer-related signaling pathways, including the PI3K-Akt signaling pathway, P53 pathway, IL2-STAT5 signaling pathway, PD-L1 expression and PD-1 checkpoint pathway in cancer, and B-cell receptor signaling pathway. STAT5 is regulated by the IL-2 family and significantly contributes to tumor cell survival and malignant progression of disease through influencing NK cell (36). P53 plays a key role in cancer-cell-autonomous functions. The loss of P53 can lead to the decrease of recruitment and activity of myeloid and T cells, and eventually result in immune evasion (37). Alissa Chackerian’s team suggested that ICD can be induced by dinaciclib and enhance anti-PD1-mediated tumor suppression (38).

Furthermore, tumor immune infiltration landscape was calculated by the ESTIMATE and CIBERSORT algorithms. The score calculated by ESTIMATE for the two subtypes revealed that the ICD-high subtype was negatively correlated with tumor purity and positively correlated with immune, stromal, and estimate scores. Thus, HLA and checkpoint genes showed considerably high expression in the ICD-high subtype.

The ICD prognostic signature was built with TF, FOXP3, LY96, SLC7A11, HSP90AA1, UCN, IFNB1, and TLR3 to predict the prognosis by quantification metric. Patients in the high-risk group had significantly poorer prognosis compared with the low-risk group according to the KM survival analysis and ROC curve, and an external dataset was introduced for validation. We evaluated and found a significant correlation between risk score and clinical factors such as grade, stage, T staging, and M staging. Moreover, CD8 T cells, follicular helper T cells (Tfh), and activated NK cells showed a negative correlation with risk score whereas M0 macrophages showed a positive correlation. Tfh cells were accepted as a distinct lineage of helper CD4 T cells. Tfh is associated with the presence of tertiary lymphoid structures (TLS), which were commonly linked to better outcome (39, 40). It was reported by Timothy W. Hand and colleagues that Tfh cells promote the formation of TLS and drive antitumor immunity in colorectal cancer (41). In addition, Julie Niogret’s team revealed that Tfh cells significantly contribute to CD8-dependent antitumor immunity and anti-PD-L1 efficacy (42). Our findings indicated that our signature was a good predictor of immunotherapy response rate. We then validated these results through TIDE analysis. A lower percentage of responders was observed in the high-risk group compared with the low-risk group. The result of submap analysis dramatically showed the better response of the high-risk group to anti-PD-1 therapy. Subsequently, we predicted the potential useful small molecular drugs through CMap analysis.

According to results of Cytoscape and supervised regression random forest algorithm, we determined LY96 (Lymphocyte antigen 96) as a hub gene to ICD in KIRC. LY96, also known as myeloid differentiation 2 (MD2), is a co-receptor to TLR4. LY96 is considered to play a key role in inflammation and immune-related diseases such as rheumatoid arthritis, Crohn’s disease, and inflammatory diabetic cardiomyopathy (43–45). Several studies have shown that LY96 is correlated with tumorigenesis and progression (46). The interaction of LY96 and TLR4 promotes the release of pro-inflammatory cytokines and adhesive molecules, which accelerates colon cancer growth and lung metastasis (47). In gastric cancer, LY96 can activate macrophage-mediated NF-κB and STAT3 pathways to promote tumor progression (48). The result of qRT-PCR validated the upregulated expression of LY96 in RCC cell lines and clinical samples. Additionally, CCK-8 and colony formation experiments demonstrated that downregulation of LY96 inhibited the proliferation ability of 786-O cells. We also validated the different expression of all signature genes in tissues.

In conclusion, our research evaluated the associations of prognosis, biological function and pathways, and immune infiltration landscape with ICD subtypes in KIRC. Furthermore, we constructed a prognosis-related ICD signature based on TF, FOXP3, LY96, SLC7A11, HSP90AA1, UCN, IFNB1, and TLR3. The signature was verified to have an independent prognostic value and provided an exact survival prediction. In addition, we determined LY96 as a potential biomarker. Based on previous studies, our research might provide a theoretical basis for the development of a novel immunotherapy for the treatment of KIRC. However, several limitations remain to be addressed in our study. The cohort in research mainly comprise Western samples, which may influence the usability of the findings to other populations. Further clinical trials were also required to verify our conclusion.

## Data availability statement

The datasets presented in this study can be found in online repositories. The names of the repository/repositories and accession number(s) can be found in the article/**Supplementary Material**.

## Ethics statement

The studies involving human participants were reviewed and approved by Department of Urology, The Second Affiliated Hospital of Nanjing Medical University, Nanjing. The patients/participants provided their written informed consent to participate in this study.

## Author contributions

SJ: Data curation and Conceptualization. YD: Writing-Original manuscript. JW: Editing Methodology. XZ: Statistical analysis and R codes. WL, YW, HZ and LS: Writing-Review. JY: Validation and Software. QZ: Project administration. All authors contributed to the article and approved the submitted version.
