# Supplementary figures and images for: Identification of immunogenic cell death-related signature on prognosis and immunotherapy in kidney renal clear cell carcinoma
Source: Front Immunol. 2023 Aug 18;14:1207061. doi: 10.3389/fimmu.2023.1207061 (PMC10472448; doi:10.3389/fimmu.2023.1207061)

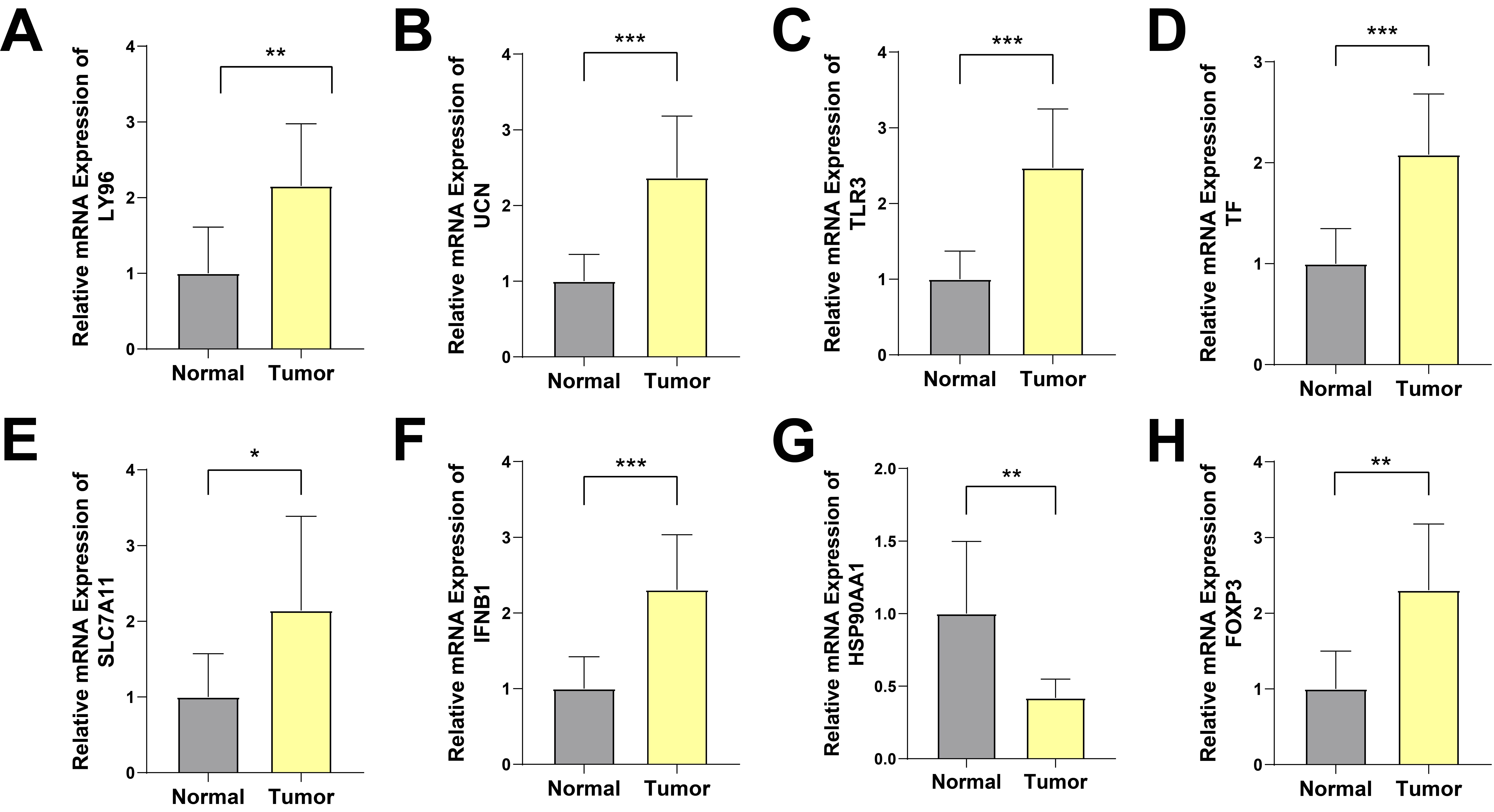

Supplement: Supplementary file 1 [file Image_1.jpeg]
