# Supplementary material for: Identification of immunogenic cell death-related signature on prognosis and immunotherapy in kidney renal clear cell carcinoma
Source: Front Immunol. 2023 Aug 18;14:1207061. doi: 10.3389/fimmu.2023.1207061 (PMC10472448; doi:10.3389/fimmu.2023.1207061)
Supplement: Supplementary file 4 [file Table_3.docx]

**Table S3. The sequences of primers used for quantative real-time PCR.**

| **Primers** | **Sequence (5’-3’)** |
| --- | --- |
| LY96 (forward) | GAAGCAGTATTGGGTCTGCAA |
| LY96 (reverse) | TTGGAAGATTCATGGTGTTGACA |
| TF (forward) | GTGTGCAGTGTCGGAGCAT |
| TF (reverse) | CATCGGATGGAATGACGCTTT |
| FOXP3 (forward) | GTGGCCCGGATGTGAGAAG |
| FOXP3 (reverse) | GGAGCCCTTGTCGGATGATG |
| SLC7A11 (forward) | TCTCCAAAGGAGGTTACCTGC |
| SLC7A11 (reverse) | AGACTCCCCTCAGTAAAGTGAC |
| HSP90AA1 (forward) | CATAACGATGATGAGCAGTACGC |
| HSP90AA1 (reverse) | GACCCATAGGTTCACCTGTGT |
| UCN (forward) | CAACCCTTCTCTGTCCATTGAC |
| UCN (reverse) | CGAGTCGAATATGATGCGGTTC |
| IFNB1 (forward) | ATGACCAACAAGTGTCTCCTCC |
| IFNB1 (reverse) | GGAATCCAAGCAAGTTGTAGCTC |
| TLR3 (forward) | TTGCCTTGTATCTACTTTTGGGG |
| TLR3 (reverse) | TCAACACTGTTATGTTTGTGGGT |
| Actin (forward) | AGTTGCGTTACACCCTTTCTTG |
| Actin (reverse) | GCTGTCACCTTCACCGTTCC |
