# Supplementary material for: Identification of immunogenic cell death-related signature on prognosis and immunotherapy in kidney renal clear cell carcinoma
Source: Front Immunol. 2023 Aug 18;14:1207061. doi: 10.3389/fimmu.2023.1207061 (PMC10472448; doi:10.3389/fimmu.2023.1207061)
Supplement: Supplementary file 5 [file Table_4.docx]

**Table S4. The sequences of siRNAs used in this study.**

| siRNA | Sequence (5’-3’) |
| --- | --- |
| LY96 siRNA | CCGAGGATCTGATGACGATTA |
| NC siRNA | TTCTCCGAACGTGTCACGT |
